# Systematic review of the treatment of moderate acute malnutrition using food products

**DOI:** 10.1111/mcn.12898

**Published:** 2019-10-30

**Authors:** Natasha Lelijveld, Alexandra Beedle, Arghanoon Farhikhtah, Eglal Elamin Elrayah, Jessica Bourdaire, Nancy Aburto

**Affiliations:** ^1^ School of Human Development and Health University of Southampton Southampton UK; ^2^ Department of Population Health London School of Hygiene and Tropical Medicine London UK; ^3^ United National World Food Programme Rome Italy

**Keywords:** acute malnutrition, food products, moderate acute malnutrition, nutrition counselling, supplementary food

## Abstract

There is currently a lack of international guidance on the most appropriate treatment for moderate acute malnutrition (MAM), and discrepancies in national treatment guidelines exist. We aimed to explore whether food interventions are effective for MAM children 6–59 months old and whether they result in better outcomes compared with no treatment or management with nutrition counselling. A systematic literature search was conducted in October 2018, identifying studies that compared treating MAM children with food products versus management with counselling or no intervention. A total of 673 abstracts were screened, 101 full texts were read, and one study was identified that met our inclusion criteria. After broadening the criteria to include micronutrients in the control group and enrolment based on out‐dated anthropometric criteria, 11 studies were identified for inclusion. Seven of these found food products to be superior for anthropometric outcomes compared with counselling and/or micronutrient supplementation; two of the studies found no significant benefit of a food product intervention; and two studies were inconclusive. Hence, the majority of studies in this review found that food products resulted in greater anthropometric gains than counselling or micronutrient interventions. This was especially true if the supplementary food provided was of suitable quality and provided for an adequate duration. Improving quality of and adherence to counselling may improve its effectiveness, particularly in food secure contexts. There is currently a paucity of comparable studies on this topic as well as a lack of studies that include important functional outcomes beyond anthropometric proxies.

**Key messages**

- There is currently a lack of analogous studies comparing the two different management approaches recommended for MAM in national guidelines: food supplementation or nutrition counselling. Studies that include functional outcomes are especially lacking.
- The evidence in this review suggests that supplementary foods do support recovery from MAM and are often more effective at promoting anthropometric recovery than nutrition counselling, with or without the addition of micronutrient supplements.
- The type and duration of supplementary food provided is important; supplementation with high‐quality protein and adequate micronutrient content, for 3 months, is recommended.
- Improving quality and adherence to counselling interventions may improve effectiveness.

## 1 INTRODUCTION

Worldwide, approximately 8% of children under 5 years of age are acutely malnourished (Development Initiatives, 2018). Children with severe acute malnutrition (SAM), defined as a weight‐for‐height *Z*‐score (WHZ) < −3, mid‐upper arm circumference (MUAC) <11.5 cm, or bilateral pitting oedema, experience an increased number of infectious diseases, delayed cognitive development, and decreased adult stature and productivity (Bhutta et al., 2017; Black et al., 2013). Since 2007, the United Nations agencies working with nutrition and health have collectively recommended home‐based therapy with ready‐to‐use therapeutic food (RUTF) for the treatment of uncomplicated SAM (World Health Organization [WHO], World Food Programme [WFP], United Nations System Standing Committee on Nutrition, & United Nations Children's Fund [UNICEF], 2007).

However, there is currently no consensus on how best to treat moderate acute malnutrition (MAM). The WHO has recognized the present lack of global guidelines for the treatment of MAM and called for more evidence in this area to inform related policies (WHO, 2017). Additionally, at national level, there are discrepancies in treatment strategies for MAM, if any are present at all. We know that children defined as MAM (WHZ ≥ −3 and <−2 and/or MUAC ≥11.5 and <12.5 cm) are at higher risk of mortality, morbidity, and deteriorating into SAM; hence, finding an effective method of supporting this group is crucial for meeting the second Sustainable Development Goal of achieving zero hunger by 2030 and ending all forms of malnutrition (James et al., 2016; L. M. Lenters, Wazny, Webb, Ahmed, & Bhutta, 2013).

Certain national guidelines for MAM treatment suggest the provision of supplementary food products, whereas others state that caregivers of MAM children should be provided with nutrition counselling alone. There is some debate about the necessity of supplementary foods for MAM: Do they result in better outcomes than no treatment or management with nutrition counselling? With the rise of noncommunicable diseases in low‐income settings and our current lack of understanding of the exact causes, we need to be confident that MAM interventions are optimizing immediate survival as well as long‐term health (Fabiansen et al., 2017; Shrimpton & Rokx, 2012; WHO, 2014). Moreover, food product interventions can be costly and lack sustainability; hence, their effectiveness should be established with concrete evidence and the outcomes of children managed with alternative methods assessed.

This review therefore aimed to identify and to synthesize the current evidence on outcomes of MAM children treated with food interventions compared with no treatment or management with nutrition counselling. Through identifying the current state of knowledge and highlighting evidence gaps, we hope to inform future research and international guidelines for the treatment of MAM.

## 2 METHODS

### 2.1 Search strategy

The systematic literature search was conducted on October 22, 2018 in Pubmed, Cochrane, and ScienceDirect databases, as well as resources catalogued on the following websites: Emergency Nutrition Network, Valid International, Evidence Aid, and State of Acute Malnutrition. Reference lists of eligible publications were also searched for relevant titles. Both peer‐reviewed publications and grey literature were eligible for inclusion. The search term strategy and eligibility criteria were guided by the “Population, Interventions, Control, and Outcome (PICO) framework” presented in Table 1.

**Table 1:** Population, interventions, control, and outcome framework for search strategy

Population	Intervention	Comparison	Outcome
Children with MAM (6–59 months) diagnosed by WHO growth standards using MUAC ≥11.5 to <125 cm and/or WHZ ≥ −3 to <−2, or WFH ≥ 70% to <80% of the median NCHS growth references Absence of bilateral pitting oedema	Ready‐to‐use supplementary foods (RUSF) Lipid‐based Nutrient Supplements (LNS) Fortified blended foods such as Supercereal Plus Ready‐to‐use therapeutic foods (RUTF) Other macronutrient food supplements	Nutrition counselling alone No intervention	Recovery Weight gain MUAC improvement Nonrecovery/Nonresponse Default Deterioration into SAM Relapse Death Length of stay Tolerance and acceptability Morbidities

The following search terms were used: “Moderate acute malnutrition” or “untreated MAM” or “moderately malnourished children” or “supplementary food” or “RUSF,” including Medical Subject Headings and free text terms, restricted to “human studies” and “children: 0–18 years” where search function allowed. No restrictions on publication date and language were applied.

### 2.2 Screening and study selection

Two researchers conducted the screening process independently (EEE and AB); titles and abstracts of all search results were screened, guided by the PICO framework. Potentially eligible full texts were independently assessed for inclusion and a discussion between reviewers then defined the final results. Data were extracted from the included texts using a standardized form. Risk of bias was assessed at study level using the quality appraisal checklist from the National Institute for Health and Clinical Excellence public health guidelines (Higgins & Green, 2011; National Institute of Health and Care Excellence, 2012). A meta‐analysis of recovery rates was planned; however, heterogeneity of methods and outcomes as well as limited number of eligible studies prevented this. The Preferred Reporting Items for Systematic Reviews and Meta‐Analyses checklist was followed for reporting results (Moher et al., 2015).

## 3 RESULTS

**Figure 1:**
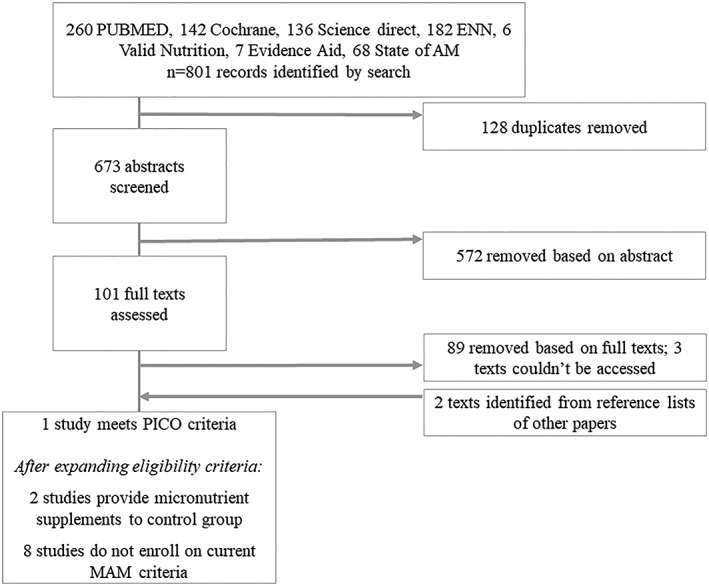
Search results flow diagram. MAM, moderate acute malnutrition; PICO, Population, Interventions, Control, and Outcome framework

We screened 673 abstracts, ultimately identifying only one study that strictly meets the predefined PICO framework (see Search result flow diagram in Figure 1). Due to this very limited number of eligible studies, we discussed widening the inclusion criteria and identified two studies that provided micronutrient supplementation to the control group and eight studies that did not enrol children based on current, common definitions of MAM; however, MAM children were part of the sample, for example, enrolment based on low weight‐for‐age or MUAC < 12.9 cm.

Ten of the studies were randomized controlled trials; the other one was a prospective cohort study. A summary of the results is presented in Table 2. Seven of the studies found food products to be superior with regard to anthropometric outcomes (weight, height, or MUAC), compared with nutrition counselling and/or micronutrient supplementation; two of the studies found no significant benefit of a food product intervention compared with control; and two of the studies were not conclusive either way. A more detailed summary of the studies including sample size, setting, and a summary of the outcomes is presented in Table A1, and a summary of the internal and external risk of bias assessment based on National Institute for Health and Clinical Excellence quality appraisal checklist is in Table A2.

**Table 2:** Summary of review results

Author, year, study design	Location, sample size, target age, and admission criteria	Intervention treatment	Control treatment	Food product better than control?	Risk of bias score^§^
(Nikièma et al., 2014) *Cluster RCT*	Burkina Faso *N* = 1,974 6–24 months, WHZ < −2 and ≥3	Locally produced RUSF Supercereal Plus	Child‐centred counselling (CCC)	Yes, better anthropometric recovery due to lower default	++/+
Micronutrients provided to control groups					
(M. Hossain et al., 2012; M. I. Hossain & Ahmed, 2014; M. I. Hossain & Yasmin, 2016) (conference abstracts) *Cluster RCT*	Bangladesh *N* = 227 6–24 months, WHZ < −2 and ≥−3	Cereal‐based supplement (SF) Cereal supplement and psychosocial stimulation (SF + PS)	Health education and micronutrients at hospital (HC) Health education and micronutrients at clinic (CC) Psychosocial stimulation (PS)	Maybe, Not possible to distinguish between benefits of supplement versus psychosocial stimulation	−/−
(Javan et al., 2017) *RCT*	Iran *N* = 70 9–24 months, WHZ <−2 & ≥−3 and referred for treatment	Blended flour supplementary food (chickpea, rice, wheat, barley, sugar) + multivitamins + nutritional counselling (SF)	Multivitamins + nutritional counselling (C)	Yes, better recovery, weight gain and WLZ gain	++/++
Not recruited based on current mam definitions					
(van der Kam, 2017) *RCT*	Nigeria N = 2,213 (25% of sample had MAM at enrolment) 6–59 months, Diagnosed with malaria, diarrhoea, or LRTI	RUTF, one sachet per day	1.Micronutrients, two sachets/d (MNP) 2.No supplement (C)	**No** – incidence of SAM was same for RUTF group to MNP group and no supplement group.	++/+
(Roy et al., 2005) *Cluster RCT*	Bangladesh *N* = 282 6–24 months, Weight‐for‐age 61% ‐ 75% of median (NCHS)	Intensive nutrition education + supplementary feeding (INE + SF)	Standard nutrition education (C) Intensive nutrition education (INE)	Yes, better immediate and sustained recovery	++/+
(Fauveau et al., 1992) *RCT*	Bangladesh *N* = 134 6–12 months, MUAC > 11.0 and <12.9 cm, and living in bamboo structure	Supplementary food (rice, wheat, lentils, and oil; SF)	Nutrition education (C)	Maybe, food group have larger weight gain in first 3 months but not whole 6 months	++/+
Not recruited based on current mam definitions and micronutrients provided to control groups					
(M. I. Hossain et al., 2011) *RCT*	Bangladesh N = 507 (81% of sample had WHZ <−2 at baseline) 6–24 months, WAZ < −3 (NCHS) and recovered from diarrhoea at the hospital	Health education and micronutrients at clinic + cereal‐based supplement (C–SF) Health education and micronutrients at clinic + cereal supplement and psychosocial stimulation (C–SF + PS)	1.Health education and micronutrients at hospital (HC) 2.Health education and micronutrients at clinic (CC) 3. Health education and micronutrients at clinic + psychosocial stimulation (C–PS)	Yes, better WLZ and LAZ gain.	++/+
(Heikens et al., 1989) *RCT*	Jamaica *N* = 82 3–36 months, WAZ < 80% of median (NCHS)	High energy supplement for 3 months plus weekly home visits and micronutrient supplements for 6 months (HES)	Home visits and micronutrient supplements for 6 months (HV)	Yes, better WAZ after 3 months but no difference after 6 months. But better HAZ after 6 months	+/+
Preventative trials: majority adequately nourished children in sample					
(Schlossman et al., 2017) *Pilot cluster‐ RCT*	Guinea Bissau *N* = 681 6–59 months, WHZ < 2 or WAZ < 1 or HAZ < 2	RUSF with 15% protein RUSF with 33% protein	No intervention (C)	No, controls improved an equal extent to food group	+/−
(Christian et al., 2015) *Cluster RCT*	Bangladesh *N* = 5,421 6 months, All infants in the catchment area	RUSF–R, rice‐lentil based RUSF–C, chickpea based RUSF–S, soy based Wheat–soy‐blend++ (WSB)	Nutrition counselling (C)	Yes, for RUSF‐S, No benefit of WSB++ over counselling	++/++
(Grellety et al., 2012) *Prospective cohort*	Niger *N* = 2,238 (18% of sample WHZ < −2) 6–23 months, All children 60–80 cm length	1. RUSF–soy (LNS–MQ)	No supplementation (failed to register; C)	Yes, better MUAC and WLZ gain and lower mortality rate	+/−

*Note*. Risk of bias score is presented as internal/external score; (−) Poor quality, (+) Adequate quality, (++) good quality. See “WFP Specialized Nutritious Food Sheet” for detailed definitions of common supplements (WFP, 2018); see individual papers for full details of nutrient content of each supplement.

Abbreviations: LRTI, lower respiratory tract infection; WHZ, weight‐for‐height *Z*‐score; WAZ, weight‐for‐age *Z*‐score; RUSF, ready‐to‐use supplementary food; CSB++, micronutrient fortified corn–soy‐blended flour, now commonly termed “Supercereal Plus” (UNICEF, 2016); RCT; randomized controlled trial; LNS–MQ, lipid‐based nutrient supplement medium quantity.

**Table A1:** FULL SUMMARY OF LITERATURE REVIEW RESULTS

Author, year, study design	Setting and sample size	Admission criteria	Intervention treatment	Control treatment	Length of int	Outcomes reported	Food better than control?	Validity score^α^
Nikièma et al., 2014 Cluster RCT	Burkina Faso *N* = 1,974	Aged 6–24 months WHO, 2006 (WHZ > −2 and ≥−3)	Locally produced RUSF 2. Supercereal Plus (SC+)	Child‐centred counselling (CCC)	3 m	Recovery: RUSF 74%, SC+ 75%, CCC 58% *p* < .0001 Default: RUSF 7%, SC+ 4%, CCC 19% *p* < .003 SAM: RUSF 8%, SC+ 10%, CCC 12% *p* < .05 When restricted to nondefaulters, recovery = 71%, 78%, and 80% for CCC, SC+, and RUSF.	Yes	++/+
Micronutrients provided to control groups								
Hossain, Ahmed, & Brown, 2012, 2014, 2016 (conference abstracts) Cluster RCT	Bangladesh *N* = 227	Aged 6–24 months WHZ < −2 to −3 (WHO, 2006)	Cereal‐based supplement (SF) Cereal supplement and psychosocial stimulation (SF + PS)	1.Health education and micronutrients at hospital (HC) 2.Health education & micronutrients at clinic (CC) 3.Psychosocial stimulation (PS)	3 m	Follow‐up attendance and gain in weight and length were greater in groups SF, SF + PS, and PS than CC and HC.	Maybe: Not possible to distinguish between benefits of supplement versus psychosocial stimulation	−/−
Javan et al., 2017 RCT	Iran *N* = 70	Aged 9–24 months with WLZ < −2 and ≥−3 and referred for treatment	Blended flour supplementary food (chickpea, rice, wheat, barley, and sugar) + multivitamins + nutritional counselling (SF)	Multivitamins + nutritional counselling (C)	3 m	Recovery rate: SF 68%, C 32% *p* = .001 Weight gain (g): SF 0.81, C 0.55, *p* = .002 WLZ gain: 0.36, C 0.02 *p* = .003	Yes	++/++
Not recruited based on current MAM definitions								
van der Kam et al., 2016 RCT	Nigeria *N* = 2,213 25% of sample had MAM at enrolment	Aged 6 to 59 months and diagnosed with malaria, diarrhoea, or LRTI MAM= WHZ <−2 and >−3, and MUAC >115 mm	RUTF, one sachet per day	micronutrients, two sachets/d (MNP) No Supplement (C)	14 days	Incidence rate of SAM in MAM children: RUTF 0.70, MNP 0.71, C 0.71 p > .05 for RUTF versus MNP and RUTF versus C	No: Incidence of SAM was same for RUTF group to MNP group and no supplement group.	++/+
Roy et al., 2005 Cluster randomised trial	Bangladesh *N* = 282	Aged 6–24 months, WA 61– 75% of median NCHS	Intensive nutrition education + supplementary feeding (INE + SF)	Standard nutrition education (C) Intensive nutrition education (INE)	3 m	Recovery rate (WAM): INE + SF 47%, INE 37%, C 18% *p* < .001 Recovery 6 months after end of intervention (WAM): INE + SF 86%, INE 59%, C 30% *p* < .0001	Yes	++/+
Fauveau et al., 1992 RCT	Bangladesh *N* = 134	Aged 6 to 12 months, MUAC > 110 and <129 mm, living in bamboo structure	Supplementary food (rice, wheat, lentils, and oil; SF)	Nutrition education (C)	6 m	Monthly weight gain in first 3 months: SF 205 g, C 159 g *p* < .05 Monthly weight gain in 6 months: SF 179 g, C 128 g *p* > .05 No significant difference in diarrhoea or other morbidities.	Maybe: Food group have larger weight gain in first 3 months but not whole 6 months	++/+
Not recruited based on current mam definitions and micronutrients provided to control groups								
Hossain, Nahar, Hamadani, Ahmed, & Brown, 2011 RCT	Bangladesh *N* = 507 81% of sample had WLZ < −2 at baseline	Aged 6‐24 months and WAZ <−3 and recovered from diarrhoea at the hospital (NCHS) Results stratified by WLZ <−2	Health education and micronutrients at clinic + cereal‐based supplement (C–SF) Health education and micronutrients at clinic + cereal supplement and psychosocial stimulation (C−SF + PS)	Health education and micronutrients at hospital (HC) Health education and micronutrients at clinic (CC) Health education and micronutrients at clinic + psychosocial stimulation (C–PS)	3 m	Whole sample: Weight gain (kg): HC 0.60, CC 0.79, C–PS 0.83, C–SF 0.92, C–SF+PS 0.90 (SF versus no SF p=0.009) Severe illness rate: No significant difference Attendance at 5^th^ visit: 54% with SF, 40% without SF For those with WLZ < −2 WLZ gain: HC 0.65, CC 0.65, C–PS 0.87, C–SF 0.94, C−SF + PS 1.19 LAZ gain: HC −0.41, CC −0.29, C−PS −0.33, C−SF −0.20, C−SF + PS −0.15	Yes	++/+
Heikens, Schofield, Dawson, & Grantham‐McGregor, 1989 RCT	Jamaica *N* = 82	Aged 3–36 months and <80% WAZ using NCHS	High energy supplement for 3 months plus weekly home visits and micronutrient supplements for 6 months (HES)	Home visits and micronutrient supplements for 6 months (HV)	3m	WAZ after 3 months: HES −2.6, HV −3.1, *p* = .007 WHZ and WAZ after 6 months: not significantly different between HES and HV groups HAZ after 6 months: HES −2.1, HV −2.7, *p* = .03	Yes, marginally	+/+
Preventative trials: majority adequately nourished children								
Schlossman et al., 2017 Pilot cluster RCT	Guinea Bissau *N* = 681	Children aged 6–59 months with WHZ < 2 or WAZ < 1 or HAZ < 2 and their mothers	RUSF with 15% protein RUSF with 33% protein	No intervention (C)	3 m	Infants 6–23 months: Change in WHZ: RUSF33 0.28, RUSF15 0.12, C 0.26 *p* > .05 Change in MUAC: RUSF33 0.62, RUSF15 0.46, C 0.28 *p* > .05 Haemoglobin: RUSF33 0.71, RUSF15 0.87, C 0.06 *p* > .05 Retinol‐binding protein: RUSF33‐0.05, RUSF15‐0.09, C 0.05 *p* > .05	No: Controls improved an equal extent to food group.	+/−
Christian et al., 2015 Cluster RCT	Bangladesh *N* = 5,421	All infants aged 6 months in the catchment area	RUSF–R, rice–lentil based RUSF–C, chickpea based RUSF–S (soy based) Wheat–soy‐blend++ (WSB)	Nutrition counselling (C)	12 m	At 18 months Prevalence stunting: RUSF–R 44%, RUSF–C 39%*, PD 40%*, WSB 44%, C 44% Prevalence wasting (WLZ): RUSF–R 16%, RUSF–C 16%, RUSF–S 14%*, WSB 18%, C 16% Prevalence underweight: RUSF–R 39%, RUSF–C 35%*, RUSF−S 33%*, WSB 40%, C 39% (*p < .05 when compared with control)	Yes for RUSF−S, No benefit of WSB++ over counselling	++/++
Grellety et al., 2012 Prospective cohort	Niger *N* = 2,238 18% of sample WLZ < −2	All children 60–80 cm length (approx. age 6−23 months) 18% of sample WLZ < −2	RUSF–soy (LSN–MQ)	No supplementation (failed to register; C)	4 m	MUAC gain (mm): LNS–MQ −2.8, C −4.0 *p* = .002 WLZ gain: LNS–MQ −0.2, C −0.3 *p* = .006 Rate of wasting per 100 child‐months: LNS–MQ 3.9, C 4.2 *p* = .05 Rate of stunting per 100 child‐months: LNS–MQ 26.8, C 14.4 *p* = .87 Mortality rate per 100 child‐months: LNS–MQ 1.6, C 2.4 *p* = .03	Yes	+/−

*Note*.^.^ Risk of bias score is presented as internal/external score; (−) Poor quality, (+) Adequate quality, (++) good quality. See “WFP Specialized Nutritious Food Sheet” for detailed definitions of common supplements; see individual papers for full details of nutrient content of each supplement.

Abbreviations: LNS–MQ, lipid‐based nutrient supplement medium quantity; LRTI, lower respiratory tract infection; RUSF, ready‐to‐use supplementary food; Supercereal Plus, micronutrient fortified corn–soy‐blended flour, formally called CSB++; RCT, randomized controlled trial; WAM, weight for age median.

**Table A2:** QUALITY APPRAISAL OF PAPERS INCLUDED IN LITERATURE REVIEW RESULTS

Study	External Validity Score (Section 1)	nternal Validity Score (Section 2‐4)
Treating moderate acute malnutrition in first‐line health services: An effectiveness cluster‐randomized trial in Burkina Faso. Nikièma et al., 2014	++	+ No blinding. Greater loss to fup in control group
Community‐based follow‐up with/without food supplementation and/or psychosocial stimulation in the management of children with moderate acute malnutrition in Bangladesh Hossain et al., 2012, 2014, 2016 (conference abstracts)	‐ Children selected from those with diarrhoea only	‐ Allocation unclear. Cluster sample size n=1 per group. Details of measurements not given. Recovery rate, and *p*‐values not presented.
**Intensive Nutrition Education with or without Supplementary Feeding Improves the Nutritional Status of Moderately‐malnourished Children in Bangladesh** Roy et al., 2005	++	+ Who did randomization? W/L not present as outcome
Limited impact of a targeted food supplementation programme in Bangladeshi urban slum children Fauveau et al., 1992	++	**+** Randomisation method resulted in more malnourished children in intervention group
Effect of short‐term supplementation with ready‐to‐use therapeutic food or micronutrients for children after Illness for prevention of malnutrition: a randomised controlled trial in Nigeria van der Kam et al., 2016	++	**+** How was randomisation managed/monitored?
Effectiveness of supplementary blended flour based on chickpea and cereals for the treatment of infants with moderate acute malnutrition in Iran: A randomized clinical trial. Javan et al., 2017	++	++
Effects of community‐based follow‐up care in managing severely underweight children Hossain et al., 2011	++	+ Not blinded, doesn't report some of the listed outcomes
The Kingston Project. I. Growth of malnourished children during rehabilitation in the community, given a high energy supplement Heikens et al., 1989	+ Only referrals	+ No mention of blinding. How randomized?
A randomized controlled trial of two ready‐to‐use supplementary foods demonstrates benefit of the higher dairy supplement for reduced wasting in mothers, and differential impact in infants and children associated with maternal supplement response Schlossman et al., 2017	**+** Who selected villages?	**‐** Controls changed behaviour, underpowered, not blinded
Effect of fortified complementary food supplementation on child growth in rural Bangladesh: a cluster‐randomized trial Christian et al., 2015	++	++
Effect of mass supplementation with ready‐to‐use supplementary food during an anticipated nutritional emergency Grellety et al., 2012	+ Intervention versus control not randomized	‐ Not randomized. Not intervention. Difference in loss to follow up between intervention and control groups
*Note*.(−) Poor‐quality, (+) Adequate‐quality, and (++) Good‐quality.		

The one study that met the original PICO criteria was a cluster‐randomized controlled trial in Burkina Faso with 18 clusters across 3 study arms (Nikièma et al., 2014). MAM was defined as WHZ < −2 and ≥−3 based on WHO, 2006, reference. One arm provided a soy‐based ready‐to‐use supplementary food (RUSF); another provided fortified corn–soy‐blended flour (Supercereal Plus; UNICEF, 2016), and the control arm provided weekly personalized counselling to caregivers. The recovery rate (WHZ > −2) after 3 months in the control arm was significantly lower (57.8%) than the Supercereal Plus arm (74.5%) and the RUSF arm (74.2%; *p* < .0001). However, when a “per protocol” analysis was applied, there was no significant difference in recovery rate between the groups, suggesting that the beneficial effect of the food products was due to lower defaulting in those groups than in the control group.

Nonadherence or defaulting in the counselling/education group was also a problem for the study by Hossain et al. They found that children who attended at least five of the six follow‐up visits gained more in weight (median: 0.86 vs. 0.62 kg, *p* = .002) and length (median: 2.4 vs. 2.0 cm, *p* = .009) than those who attended fewer times (M. I. Hossain & Ahmed, 2014; M. I. Hossain & Yasmin, 2016).

Food security status also differed across the studies and could affect the results, particularly the success of counselling as treatment. The study by Nikièma et al. (2014) was conducted in a “relatively food‐secure” context. One other study states that it was conducted in a relatively food‐secure setting, taking place in an urban area of Iran (Javan, Kooshki, Afzalaghaee, Aldaghi, & Yousefi, 2017). They found food supplementation with counselling to be superior to multivitamins and counselling alone; although there was some spontaneous recovery (WHZ > −2; 32%) in the counselling group, this was much lower than in the food supplementation group (80%). Three studies mention that their study populations were likely to be food insecure. Roy et al. (2005) suggest that, although food supplementation had the best weight gain, an “intensive counselling” group still had better weight gain than the “standard counselling” group, despite low food security, whereas Christian et al. (2015) conclude that counselling alone is not sufficient in areas of food insecurity.

Of the studies that used WHZ < −2 and ≥−3 as the definition of MAM, the recovery rate was between 75% and 68% when providing a supplementary food intervention compared with counselling and/or micronutrient supplements, where recovery rate was between 58% and 32% (Javan et al., 2017; Nikièma et al., 2014).

Across the papers, there was an inference that the type of food product provided is important. The study by Fauveau et al. suggested that the lack of iron and zinc in the supplementary food was the reason for the limited longer term effect of the supplement (Fauveau et al., 1992). Additionally, Christian et al. found consistent anthropometric benefits (WHZ and HAZ) of a soy‐based RUSF as well as multiple benefits of the chickpea‐based RUSF. However, far fewer beneficial outcomes were shown for the rice–lentil‐based RUSF and no benefit at all of fortified wheat–soy‐blended flour compared with nutrition counselling (Christian et al., 2015).

Of the two studies that saw no benefit from a food product, neither targeted MAM children specifically. The study by van der Kam et al. in Nigeria enrolled children aged 6 to 59 months who were diagnosed with malaria, diarrhoea, or lower respiratory tract infection (van der Kam, 2017). The intervention group were provided with one sachet of RUTF per day for 14 days, whereas the two control groups were either provided with micronutrient powder or no supplement. Among ill children who had MAM at enrolment (WHO, 2006, WHZ < −2 and ≥−3), the incident rate of developing SAM was not significantly different between the groups. The authors speculated that 14 days of supplementation was not long enough to reduce incidence of SAM. The other study that saw no benefit from a food intervention, compared with no intervention, was conducted by Schlossman et al. in Guinea Bissau using “at risk” infants and children (Schlossman et al., 2017). This was defined as children aged 6–59 months with either WHZ < 2 or WAZ < 1 or HAZ < 2. Mean WHZ at enrolment was −1.0, suggesting that most children sampled were not MAM cases, and the results were not stratified by MAM status at enrolment. All groups saw significant improvements in weight and height. The authors suggest that improvements in the control group were due to increased focus by community health workers on nutrition recommendations as a result of the study. However, it is not possible to conclude whether or not there were anthropometric improvements in MAM children specifically, in either the control or intervention groups.

## 4 DISCUSSION

One study was found that met the original PICO criteria, comparing MAM treatment with a food product to either counselling or no intervention. After broadening the criteria to include studies that provided micronutrient supplements to the control group and/or did not use current definitions of MAM but likely included children with MAM in their sample, the review was composed of 11 studies. Seven out of 11 studies found food product interventions to be superior with regard to anthropometric outcomes compared with that of counselling and/or micronutrient supplementation; 2 out of 11 studies found no significant benefit of a food product intervention; and 2 out of 11 studies were inconclusive.

It is established that children with MAM require increased intake of energy and essential nutrients over and above those required by healthy children (L. Lenters, Wazny, & Bhutta, 2016; WHO, 2012). The evidence presented in this review suggests that food products do support recovery from MAM and are often more effective at promoting anthropometric recovery, than nutrition counselling, with or without the addition of micronutrient supplements. Although not all the studies assessed the same anthropometric measures, gains in weight (both WAZ and WHZ), height, and MUAC were all found across the publications, as well as reductions in prevalence of wasting and underweight.

Lack of adherence to counselling programmes may be one of the limitations influencing their effectiveness among control groups in these studies. The “per protocol” analysis by Nikièma et al. suggests that, if adhered to, the counselling programme may be as effective as the food intervention. Hence, finding ways to improve adherence to counselling interventions should be explored. However, it is also important to note that possible confounding factors may be influencing results, such as greater socioeconomic status, willingness to change behaviour, or greater care‐seeking behaviour among adhering mothers. Studies exploring the use of counselling would benefit from including direct measures of behaviour change, rather than just attendance at sessions.

Food security status may also be an important factor in determining the effectiveness of counselling interventions. The study by Nikièma et al. (2014) was conducted in a relatively food‐secure context, which may have added to its success in the per protocol analysis. However, the only other study to mention that it took place in a relatively food‐secure setting (urban Iran) still found food supplementation to be superior in recovering WHZ than micronutrients and counselling (Javan et al., 2017). It is generally felt that counselling alone is not sufficient in areas of food insecurity, as concluded by Christian et al. (2015).

The quality and content of nutrition counselling interventions also require consideration. A review of dietary counselling for the treatment of MAM was conducted by Ashworth and Ferguson in 2009 (Ashworth & Ferguson, 2009); 10 studies were examined; the majority had quasi‐experimental or observational study designs. The review concludes that counselling messages tended to be vague and were unlikely to be effective. They also found that it was difficult to achieve adequate zinc and vitamin E from home foods alone. In the studies they reviewed, they found that rates of weight gain tended to be slow, with estimates of 1 to 2 g/kg/day. This was likely due to repeated exposure to pathogens and high rates of stunting, as well as counselling being delivered by minimally trained personnel or volunteers. In successful programmes, frequent, regular exposure to a few simple, uniform, age‐appropriate messages, together with an opportunity for interaction between caregiver and counsellor, was found to be important.

Not all studies in our review found food supplements to be superior to nutrition counselling. A key consideration is the type of supplementary food provided, as well as the dosage and length of treatment. Studies in the review specifically highlighted the micronutrient content and protein quality of supplements as likely influential factors. The majority of studies provided supplements for at least 3 months; however, one study provided one sachet of RUTF for 14 days and was found to be ineffective at preventing SAM in MAM children recovering from illness. The health status of MAM children at admission should also be a consideration when selecting the most appropriate treatment; this may be at an individual level or at a more regional level based on morbidity rates. There are currently no international guidelines on the treatment of “complicated” MAM cases, another area which needs attention (Polnay, 2019).

For “uncomplicated” MAM cases, the United Nations WFP currently recommends the use of RUSFs or fortified blended flours for the treatment in children 6–59 months, for which a number of key ingredients are specified (UNICEF, 2016; WFP, 2018); however, many other supplementary food products exist, and the studies in this review highlight a lack of standardization of treatment products across settings. A review of studies comparing different food products for treatment of MAM was conducted in 2012 and found moderate to high quality evidence that both lipid‐based nutrient supplements and blended foods are effective in treating children with MAM (Lazzerini, Rubert, & Pani, 2013). There is also on‐going exploration of the use of RUTF to treat MAM children, in order to streamline supply chains with SAM treatment programmes, which may increase MAM treatment coverage and reduce program costs (Bailey et al., 2018; Maust et al., 2015).

The results of this review have some limitations. The heterogeneity in the definitions of MAM and recovery meant it was not possible to conduct meta‐analyses. Better standardization of nutrient content in supplementary food and nutrition counselling content for MAM treatment would also aid comparison of results in the future. Another major limitation of the current literature is the lack of studies including functional outcomes following MAM treatment. Anthropometric indicators are a proxy for health and nutrition status; however, they do not give definitive answers about a child's ability to survive and thrive in the longer term (Leroy & Frongillo, 2019). MAM intervention studies comparing outcomes such as cognitive function, school achievement, immune function, physical fitness, microbiome, and indicators of longer term noncommunicable disease risk, such as body composition and metabolomics, are very much needed.

Careful considerations should be made prior to the generalization of these results across all contexts. The studies in this review do have a broad geographical scope: Five of the studies were conducted in Bangladesh, four in West Africa, one in Jamaica, and one in Iran. However, emergency contexts are not well represented, with only one study being conducted during a nutrition emergency (Grellety et al., 2012). In addition, the level of food security, burden of infectious disease, and access to health services may also influence the generalizability of these results to all contexts.

In conclusion, there is currently a paucity of studies on this topic, which use standard definitions of MAM and recovery, as well as a lack of studies including important functional outcomes beyond anthropometric proxies. Future research is needed to address these evidence gaps. The majority of studies in this review found that food products resulted in greater anthropometric gains than counselling or micronutrient interventions. The type and duration of supplementary food provided is important, and these studies showed that supplementation with high‐quality protein and adequate micronutrient content, for 3 months, was most commonly recommended. In addition, it was suggested that improving quality and adherence to counselling interventions in food secure settings may improve their effectiveness. Policymakers should consider these factors when improving the recommendations for MAM treatment, which are urgently needed.

## CONFLICTS OF INTEREST

The authors declare that they have no conflicts of interest.

## CONTRIBUTIONS

NA and EEE developed the concept for this paper. EEE, NL, and AB conducted the research. NL wrote the draft manuscript, with input from all other authors. All authors read and approved the final manuscript.
